# Integration and Spatial Organization of Signaling by G Protein-Coupled Receptor Homo- and Heterodimers

**DOI:** 10.3390/biom11121828

**Published:** 2021-12-03

**Authors:** Roberto Maggio, Irene Fasciani, Marco Carli, Francesco Petragnano, Francesco Marampon, Mario Rossi, Marco Scarselli

**Affiliations:** 1 Department of Biotechnological and Applied Clinical Sciences, University of L’Aquila, 67100 L’Aquila, Italy; irene.fasciani@univaq.it (I.F.); francesco.petragnano@student.univaq.it (F.P.); mario.rossi@univaq.it (M.R.); 2 Department of Translational Research and New Technologies in Medicine and Surgery, University of Pisa, 56126 Pisa, Italy; carlimarco@outlook.it (M.C.); marco.scarselli@med.unipi.it (M.S.); 3 Department of Radiotherapy, “Sapienza” University of Rome, Policlinico Umberto I, 00161 Rome, Italy; francesco.marampon@uniroma1.it

**Keywords:** G protein-coupled receptor, homodimerization, heterodimerization, signal integration, signal compartmentalization

## Abstract

Information flow from a source to a receiver becomes informative when the recipient can process the signal into a meaningful form. Information exchange and interpretation is essential in biology and understanding how cells integrate signals from a variety of information-coding molecules into complex orchestrated responses is a major challenge for modern cell biology. In complex organisms, cell to cell communication occurs mostly through neurotransmitters and hormones, and receptors are responsible for signal recognition at the membrane level and information transduction inside the cell. The G protein-coupled receptors (GPCRs) are the largest family of membrane receptors, with nearly 800 genes coding for these proteins. The recognition that GPCRs may physically interact with each other has led to the hypothesis that their dimeric state can provide the framework for temporal coincidence in signaling pathways. Furthermore, the formation of GPCRs higher order oligomers provides the structural basis for organizing distinct cell compartments along the plasma membrane where confined increases in second messengers may be perceived and discriminated. Here, we summarize evidence that supports these conjectures, fostering new ideas about the physiological role played by receptor homo- and hetero-oligomerization in cell biology.

## 1. Introduction

Communication in biology is the process of moving information from a source to a receiver and, information becomes “informative” only if it can be processed in a meaningful form from the receiver. Exchange and interpretation of information is essential in biology and understanding how cells integrate signals from a variety of information-coding molecules into complex orchestrated responses is a major challenge for modern cell biology.

In complex organisms, cell to cell communication occurs mostly through neurotransmitters and hormones and the function of message recognition and transduction inside the cells is allotted to receptors. Cells express several families of receptors that can be roughly divided into three main categories: the G protein-coupled receptors (GPCRs) [1], the ligand gated ion channel receptors [2] and the enzyme-linked tyrosine kinase receptors [3]. These three receptor families subserve different functions in the cells. For example, the activation of ligand gated ion channel receptors increases the plasma membrane permeability to ions and allows a very rapid exchange of ions between the extra- and the intra- cellular environments triggering fast cell responses. In contrast, the activation of the other two receptor types modulates the intracellular levels of important metabolic components and genes transcription, thus producing slower cell responses to external stimuli. These three families of receptors were originally thought to be independent from each other; however, there is evidence that strongly suggest a broad interconnection around these receptor families including physical and functional interactions. This review mainly focuses on GPCRs and their ability to integrate stimuli, while function and interactions of the other two families of receptors will be discussed briefly.

## 2. The GPCR Signaling ‘Hardware’

GPCRs are the largest family of membrane receptors, with nearly 800 genes coding for these proteins. GPCRs cross the plasma membrane seven times and have three extracellular and three intracellular loops with an extracellular N-terminus and an intracellular C-terminus [4]. Except for the amine receptors and some other GPCRs, such as the adenosine and purinergic receptors, the extracellular part of GPCRs is deputed to agonists recognition, the transmembrane core shafts the activation signal to the intracellular portion of the receptor that is deputed to couple to intracellular transducers. The evolutionary success of GPCRs is thought to depend upon their distinctive property of being readily adaptable for new sensory functions while maintaining conserved transmembrane cores and intracellular signal transduction mechanisms [5]. In fact, their ability to be activated by such different types of stimuli like amines, photons, odorants, neurotransmitters, and hormones, resides mostly in their extracellular domains.

G proteins are the first and more characterized partners of GPCRs. G-proteins are trimeric proteins activated by the nucleotide GTP composed of α, β, and γ subunits with the α on one side and the β/γ subunits, tightly associated, on the other side, which can be regarded as two independent functional units [6]. Thirty-five genes coding for G protein subunits have so far been discovered (18α, 5β, and 12γ subunits). Based on their structural similarity and functional activities the α subunits are sub-grouped into four distinct families: (i) Gαs, whose activation stimulates the adenylyl cyclase enzyme; (ii) Gαi, which inhibits the activity of adenylyl cyclase and opens the voltage-dependent potassium channels or closes voltage-dependent Ca^2+^ channels; (iii) Gαq whose activation stimulates the phospholipase enzyme; and finally (iv) Gα12, which activates the small GTPase Rho. Gβ/γ represent additional functional elements that activate selectively different effectors [6]. It is worth mentioning that recently, it has been shown that G proteins can also be activated in a GPCR-independent way [7]. Receptor-independent activators of G-protein signaling (AGS) play surprising roles in signal processing and have opened new areas of research related to the role of G proteins in signal transduction [8].

The exclusive activation of G proteins by GPCRs is considered incomplete and would not explain the complexity of interaction between cells. Evidence has gradually emerged that GPCRs can signal through many other proteins such as β-arrestins and small G proteins, among the others [9]. Recently, using a modified membrane yeast two-hybrid approach for 48 selected full-length human ligand-unoccupied GPCRs in their native membrane environment, it has been shown that the GPCR interactome connects 686 proteins involved in a diverse range of cellular functions [10].Therefore, in addition to their well-documented and linear inward signaling through G proteins, evidence suggests that GPCRs activate more complex and branched signaling, and in the words of Kenakin [11], “receptors should be considered more akin to microprocessors than to mere on–off switches”.

G proteins do not produce second messengers per se, instead they are molecular switches within the cell that activate or inhibit enzymes, such as adenylyl cyclase [12] and phospholipase C [13]. Interestingly, genes with profound differences in the structures and domain organization encode for six distinct classes of adenylyl cyclase enzymes that catalyze the formation of cAMP, and sixteen genes encode for the phospholipase C enzymes, which catalyze the hydrolysis of lipid phosphatidylinositol(4,5)bisphosphate present in the plasma membrane into the water-soluble inositol(1,4,5)trisphosphate (IP3) and the membrane-bound diacylglycerol (DAG). The production of all these second messengers must be taken under a strict temporal and spatial control in order to allow cells to discern between the multiple incoming stimuli, and this is accomplished by buffering proteins and degrading enzymes as will be illustrated below.

Receptors, G-proteins, and enzymes that synthesize second messengers are the three key elements of the signal transduction machinery responsible for the highly efficient communication systems between cells [14].

## 3. Integration and Discrimination of Signaling in Cell Biology

**Figure 1:**
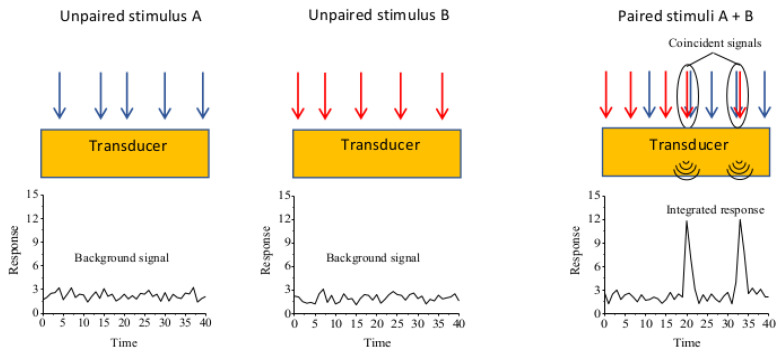
Integration of signals by temporal and spatial coincidence detection relies on separate inputs converging on a common target. Two stimuli, A and B, converging on a single transducer induce only a background response when they are unpaired. Paired arrival of these two stimuli on the target produces an integrated response when they are temporally coincident.

At any time, cells are flooded with a plethora of extracellular signals that need to be integrated and sorted out by the cell to achieve a specific physiological appropriate response. Understanding their ability to integrate and filter different stimuli represents one of the most challenging effort in cell biology. One of the most important concepts in this regard is the temporal coincidence detection (Figure 1) of separate inputs converging into a common transduction pathway.

A classic example is represented by the synergistic activation of Ca^2+^/calmodulin-stimulable adenylyl cyclase during the sensitization of the gill-withdrawal reflex in Aplysia [15]. In this sea slug, the gill-withdrawal reflex elicited by a gentle siphon touch is greatly potentiated by an unconditioned stimulus such as an electrical shock on the tail. This effect is due to the simultaneous increase in the concentration of Ca^2+^ that influxes through voltage-gated ion channels and the activation of stimulatory GαS protein by serotonin receptors. These two stimuli converge to produce a synergistic activation of Ca^2+^/calmodulin-stimulable adenylyl cyclase, thereby causing strong increases in the intracellular levels of cAMP [16,17]. Another example of this mechanism is the synergistic activation of the phospholipase Cβ by Gαq/11 and Gβ/γ proteins that results in a greater accumulation of inositol(1,4,5)trisphosphate and Ca^2+^ compared to the simple activation of Gαq/11 [18,19]. This occurs in cultured rat cortical type 2 astrocytes when there is the concomitant activation of the Gαq-coupled group 1 metabotropic glutamatergic receptor and the Gαi-coupled A1 adenosine receptor, with the latter having no effect on phospholipase Cβ stimulation per se [20]. In both cases, to boost the activation of adenylyl cyclase or phospholipase Cβ, the two converging stimuli must be temporally correlated, they must arrive timely in order to trigger a synergetic response. Furthermore, beside the temporal importance for signal integration and signal filtering, another crucial feature to be considered is where in the membrane two stimuli activate receptors within the same cells. The spatial segregation of information is fundamental for the cell and second messenger diffusion must be prevented. For example, cAMP remains confined in precisely controlled intracellular domains [21]. Likewise, the proteins that transduce and integrate different signals must be compartmentalized either by forming macromolecular complexes or by interacting with anchoring or scaffolding proteins. An example of macromolecular complex is the subtype 5 adenylyl cyclase functionally pre-coupled in a complex with the adenosine A2A and dopamine D2 receptor and their cognate Gs and Gi proteins [22]. For the second mechanism, A-kinase anchoring proteins play crucial roles in regulating compartmentalization of multi-protein-signaling networks by binding components upstream and downstream of cAMP production, including GPCRs, cAMP-dependent Rap-exchange factors, and phosphodiesterases [23,24].

## 4. Dimerization of GPCRs

Over the last two decades, much evidence accumulated suggesting that GPCRs may also physically interact with each other and function as dimers or larger order oligomeric complexes [25]. Indirect evidence of GPCR interaction was initially provided by Limbird et al. (1975) [26] for β-adrenergic receptors in frog erythrocyte membranes by a direct kinetic method. They showed that the dissociation of the radiolabeled alprenolol bound to β-adrenergic receptor was accelerated by an excess of unlabeled alprenolol, indicating the existence of site-to-site negative cooperativity among the β-adrenergic receptors. More direct evidence of physical interaction among receptors was provided by Maggio et al. (1993) [27] that showed how two reciprocal M3 muscarinic and α2 adrenergic chimeras could rescue muscarinic and adrenergic binding and function upon co-transfection by exchanging their cognate parts. Definitive prove of the existence of receptor dimerization came from bioluminescence [28] and fluorescence [29] resonance energy transfer (RET) and single-molecule microscopy detection experiments [30,31,32]. The RET methods are based on the principle that energy can be transferred from a donor to a light sensitive acceptor only if they are very close to each other. Thus, only when two GPCRs, one carrying the donor and the other the acceptor, dimerize, the energy can be transferred from one protein to the other. Single Molecule Localization Microscopy (SMLM) techniques such as Photo-Activation Localization Microscopy (PALM) and Stochastic Optical Reconstruction Microscopy (STORM) have provided extraordinary tools to directly visualize GPCRs at the single-molecule level overcoming the resolution limit imposed by the diffraction of light. These techniques have revealed the transient nature of GPCR dimers, their rapid association and dissociation kinetics, and the ligand influence on dimer formation [33,34].

Several experiments have unambiguously demonstrated that, in strictly controlled conditions, a GPCR monomer is sufficient to activate the G-protein [35,36,37,38] suggesting that dimerization does not impart the function to the receptor but only modulate its function. In this scenario, homo-dimerization modulates the strength of the receptor function, G-protein or β-arrestin coupling, and intracellular trafficking [39], while hetero-dimerization can also instruct the interacting receptors to change their signaling pathways, for instance by activating different G-proteins [40].

On a higher level of complexity, GPCRs have been shown to form higher order homo- and hetero-oligomers that could be also implicated in signal compartmentalization [41].

## 5. Signaling Potentiation by GPCR Heterodimers

**Figure 2:**
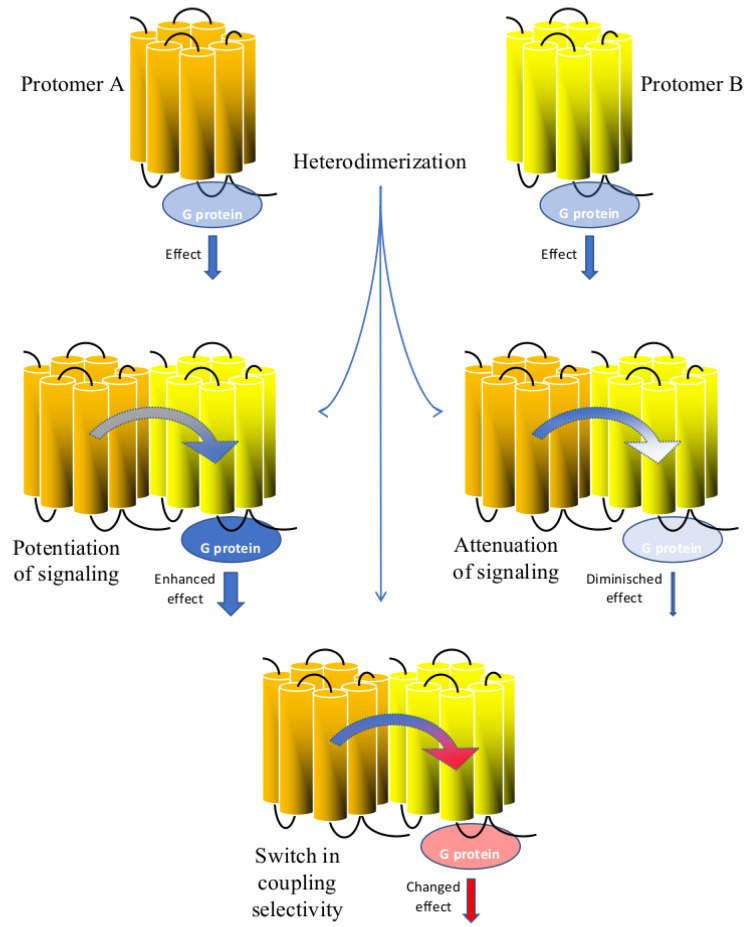
Signal integration of the GPCR heterodimer. Protomers A and B bind and activate the G protein when alone. Interaction between these two protomers leads to the formation of a heterodimer in which only one protomer binds the G protein. Heterodimerization can result in potentiation of G protein coupling and enhancement of the downstream effect or in alternative in reduction in the G protein coupling and weakening of the downstream effect. Furthermore, heterodimerization can lead to a switch in G protein coupling and change in the production of second messengers.

Arising evidence on GPCR heterodimerization strongly suggests that heterodimers might play a major role in signal integration [42] with mechanisms such as signaling potentiation (Figure 2).

As early as 1999, Jordan and Devi [43] showed that the simultaneous stimulation of κ and δ-opioid receptors with their respective agonists results in a strong synergistic activation of ERK1/2. Synergism was also reported to occur between α1A and α1B adrenergic receptors, as cells co-expressing these receptors and stimulated with phenylephrine activate ERK1/2 with higher efficacy than cells expressing either receptor alone [44]. Furthermore, synergism in second messenger activation was also reported for M2 and M3 receptors [45,46] and D2 and D3 receptors [47]. Synergistic activation of second messengers was described not only for highly homologous receptor subtypes that are stimulated by the same endogenous ligands, as in the examples reported above, but also with distantly related receptors. For instance, concomitant agonist stimulation of adenosine A2A and metabotropic glutamatergic mGluR5 receptors, which belong to GPCR families with a low sequence homology, leads to a synergistic activation of ERK1/2; on the other hand, the stimulation of each individual receptor with the respective agonist showed a very low effect on ERK phosphorylation [48]. Moreover, signal potentiation has been observed with the activation of Angiotensin II type 2 (AT2) and bradykinin B2 receptor heterodimers in relation to the nitric oxide (NO) production [49]. Another example of heterodimer induced synergism has been reported for the δ-opioid and the M3 muscarinic receptors in SH-SY5Y cells on the production of intracellular free calcium [50]. In these cells, the δ-opioid agonist [d-Pen2,5]-enkephalin largely increases and potentiates the elevation of intracellular Ca^2+^ induced by carbachol or oxotremorine-M upon activation of the M3 receptors, while having no effect by itself. We could define these enhancing effects of the heterodimers on second messenger production as a form of signal integration that plays a crucial role in the fine regulation of physiological processes.

## 6. Signaling Attenuation by GPCR Heterodimers

Another consequence of heterodimerization could be instead a synergistic attenuation of signals due to a variable coupling efficacy to G-proteins (Figure 2). One of the most investigated heterodimers whose functions are associated with a signal dampening is the μ-opioid and cannabinoid CB1 heterodimer. These studies were conducted in transfected cells, in SK-N-SH neuroblastoma cells endogenously expressing these receptors, and in the striatum where these two receptors are colocalized [51]. In particular, the simultaneous stimulation of μ and CB1 receptors reduces the signal generated by the stimulation of each receptor alone with the respective ligands. Importantly, this dampening effect was also shown in SK-N-SH cells and in the striatum by Rios et al. (2006) [51] using [35S]-GTPγS binding assays that directly measure the coupling efficiency between GPCRs and the G proteins. Taken together these data suggest that the heterodimer induced dampening of the stimulation signals was due to conformational changes in the two receptors resulting in a reduction in the G protein-coupling efficacy rather than an effect occurring downstream the GPCR heterodimer activation. Notably, Rios et al. (2006) [51] showed that the dampening effect of the integrated signals due to μ-CB1 heterodimers might impact negatively on neurites outgrowth in Neuro-2A cell lines engineered to express both μ and CB1 receptors indicating the high physiological relevance of this interaction. Another good example of reciprocal negative influence occurs in the heterodimer formed by the α2A adrenergic and the μ-opioid receptors, where the efficacy of noradrenaline to mediate ERK1/2 phosphorylation markedly decreased when morphine was coapplied to HEK293 cells co-expressing α2A adrenergic and μ-opioid receptors [52]. Remarkably, while noradrenaline and morphine applied to cells expressing individually α2A adrenergic and μ-opioid receptors, induced a concentration dependent increase in ERK1/2 phosphorylation, co-application of 10 nM noradrenaline with increasing concentrations of morphine in cells co-expressing α2A adrenergic and μ-opioid receptors, reversed the trend of the curve in a descending inhibitory way. These examples highlight how hormones and neurotransmitters binding to the protomers in a heterodimer can fine-tune their reciprocal responses in both ways, potentiating or attenuating them.

## 7. Switch in Coupling Selectivity by GPCR Heterodimers

The most convincing evidence of signaling integration at the GPCR heterodimer level is provided by the acquisition of a new coupling selectivity by the two interacting receptors (Figure 2). Change in coupling selectivity by receptor heterodimer has been shown for the first time by George et al. (2000) [53] for μ and δ-opioid receptors. In COS-7 cells transiently co-transfected with μ and δ, the stimulation with their respective agonists DAMGO and DPDPE modifies their coupling selectivity from a pertussis toxin-sensitive Gαi/Gαo protein to a pertussis insensitive Gαz protein. Likewise, Mellado et al. (2001) [54] showed that chemokine CCR2b and CCR5 receptors acquire selectivity for Gα11 protein when co-transfected in HEK293 cells and stimulated with their respective agonist monocyte chemotactic protein-1 and RANTES. In another example, Lee et al. (2004) [55] showed how dopamine D1 and D2 receptors, transiently co-transfected in COS-7 cells became selective for Gαq when the two receptors were co-stimulated, whereas the original coupling selectivity for Gαi and Gαs proteins was maintained if the stimulation of the two receptors was asynchronous.

Considering receptors belonging to different GPCR subfamilies, co-stimulation of the two protomers of the heterodimer composed by cannabinoid CB1 and dopamine D2 receptors switches G-protein coupling preferences from Gαi to Gαs [56]. Furthermore, heterodimerization of the Gαi/o coupled δ-opioid and Gαq coupled sensory neuron-specific receptor-4 (SNSR-4) did not affect the G-protein coupling of each individual receptor when they were stimulated a part. Conversely, the simultaneous activation of the two protomers by the two receptor-specific agonists, led to a switch in signaling toward the Gαq-mediated signaling of the SNSR-4 receptor, and a suppression of the Gαi/o-mediated effect [57]. In this last example, the signaling switch consists in the predominance of the coupling selectivity of one protomer over the other.

Switch in coupling selectivity challenges the classical concept of receptor/G protein interaction, suggesting that when receptors form homo or heterodimers the 1/1 stoichiometry changes to a 2/1 stoichiometry. This view is supported by several evidence, on the three-dimensional structure of GPCRs. Based on these structural data, Palczewski et al., (2000) [58] for instance, showed that the surface area of a rhodopsin dimer facing the cytosol can accommodate only a single G protein molecule. In line with this hypothesis, Baneres and Parello (2003) [59] proved unambiguously that one G protein trimer binds to a leukotriene B4 (LTB4) receptor BLT1 dimer (2xBLT1.LTB4) forming a stoichiometrically defined (2xBLT1.LTB4)Gαi2b1c2 pentameric assembly. Similar conclusions were drawn by Chinault et al. (2004) [60] who demonstrated that the yeast oligomeric α-factor receptors activate in concert only one G protein. Similarly, Herrick-Davis et al. (2005) [61] demonstrated that 5-HT2C receptor dimers bind two ligand molecules and one G protein. More recently, Liu et al. (2017) [62] studying the prototypical metabotropic glutamate receptor dimers, demonstrated that G protein activation is exclusively mediated by mGluR4 in the mGluR2-mGluR4 heterodimers, and the extracellular domains of both receptors contribute to such asymmetric transduction. mGluR2 heptahelical domain concurs in the activation of the G-protein if either the mGluR2 is occupied by a positive allosteric modulator or if mGluR4 is inhibited by a negative modulator. These data strongly indicate an asymmetry in the control of the G protein by the mGluR2-mGluR4 heterodimer, but most compelling they suggest that this asymmetry could be controlled by allosteric modulators.

## 8. Cross Talk between GPCRs and Ionotropic or Tyrosine Kinase Receptors

Signal integration is further complicated by the interactions of membrane GPCRs and the ionotropic [63] and tyrosine kinase [64] receptors. Notably, BRET and functional assays have shown a direct interaction between the glutamatergic mGluR5 and the ionotropic N-methyl-d-aspartate (NMDA) receptor in hippocampal neurons [65,66]. The interactions resulted in bidirectional inhibition with the mGluR5 receptor decreasing NMDA receptor mediated currents, and reciprocally, the NMDA receptors reducing the ability of the mGluR5 to induce intracellular calcium. Another well characterized interaction is between the dopamine D3 receptor and the nicotinic acetylcholine receptor (nAChR) that was shown by BRET studies, where the D3 receptor directly and specifically interacted with the β2 subunit of the nAChR [67]. The D3-nAChR complex was identified in cultured dopamine neurons and in mouse substantia nigra/ventral tegmental area, where it was shown to be responsible for mediating the neurotrophic effects of nicotine. Recently, a set of three small molecules targeting the D3-nAChR heteromer was synthesized that incorporate, by means of a partially rigidified spacer of variable length, the pharmacophoric substructure of the known β2-subunit-containing nAChR agonist (A-84543) and the D2/D3 agonist ropinirole [68]. The ligand named HyNDA-1, which is characterized by the shortest linker moiety, significantly modulated structural plasticity in both mice and human dopaminergic neurons in vitro. Importantly this effect was strongly counteracted by the co-incubation with either nAChR or D3 receptor antagonists.

Moreover, GPCRs also interact with tyrosine kinase receptors. One of the first evidence of this property goes back to the work of Daub et al. (1996) [69] which describes for the first time that the epidermal growth factor receptor (EGFR) was rapidly phosphorylated as a consequence of the endothelin receptors stimulation. This evidence strongly suggested that the endothelin receptor transactivates the EGFR. Strikingly, in recent years a growing set of evidence have consistently reinforced the concept that GPCRs transactivation of tyrosine kinase receptors plays crucial role in regulating physiological processes [70]. Receptor transactivation could also be bidirectional as shown for the lysophosphatidic acid receptor 1 and EGFR that could reciprocally be transactivated to promote cell proliferation in prostate cancer cells [71]. Interestingly, formation of tyrosine kinase receptor/GPCR complexes can also alter the GPCR coupling properties, such as for the cannabinoid CB2 receptor that after complexing with the human epidermal growth factor receptor 2 switches its coupling specificity from the preferred Gq/11 to the Gi or Gz protein subtype [72]. Finally, it is worth mentioning the interaction between the adenosine A2A receptor and the fibroblast growth factor (FGF) receptor [73]. In this heterodimer, the concomitant activation of the two receptors induces a robust activation of the MAPK/ERK pathway, cell differentiation and neurite extension of PC12 cells, and spine morphogenesis in primary neuronal cultures, while no effect is registered after the activation of either of the receptors alone. Furthermore, coactivation of A2A and FGF receptors was necessary for the induction of long-term memory, a form of neuronal plasticity, after pairing high frequency stimulation of glutamatergic synapses with postsynaptic depolarization [73].

## 9. GPCR Oligomerization and Spatial Compartmentalization of Signaling

**Figure 3:**
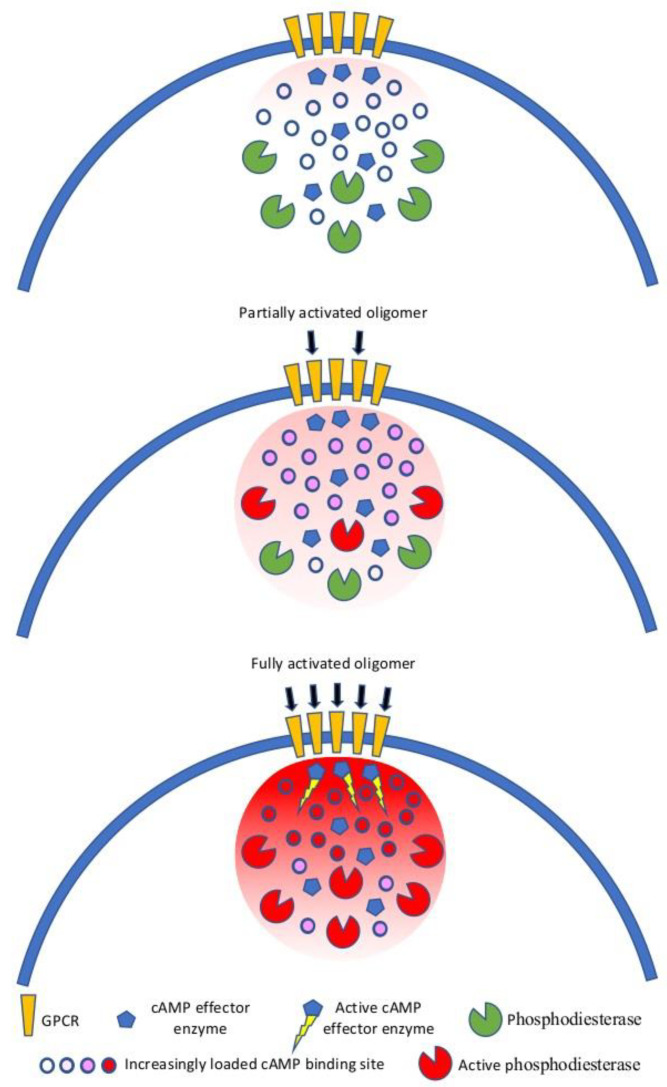
Creation of nanometer size domain of cAMP beneath a GPCR oligomer. Under basal level (upper panel) cAMP concentrations are maintained low by the presence of a large number of cAMP binding sites. Upon partial stimulation of the GPCR oligomer (middle panel), cAMP binding sites buffer the concentration of cAMP preventing it to rise above the threshold of activation of the cAMP effector enzymes. Upon full stimulation of the GPCR oligomer (lower panel), cAMP binding sites become progressively saturated, eventually leading the concentration of cAMP to rise above the threshold level of activation of the cAMP effector enzymes. The concentration gradient of free cAMP is nevertheless low enough to enable phosphodiesterases to break down cAMP and maintain it within nanometer-sized domains [21].

Cell-to-cell communication relays on hundreds of extracellular signals that impact on few intracellular second messengers. Spatial compartmentalization reduces signal redundancy at the intracellular level and enhances specificity and efficacy of signal transduction (Figure 3). 

For instance, cAMP is predominantly bound to cAMP binding sites at physiological concentrations and thus immobile. Once the adenylyl cyclase is activated, because of Gαs protein stimulation, cAMP level increases, but the binding sites buffer its free diffusion up to saturation. In this condition, with a large fraction of cAMP being buffered, degradation of cAMP by phosphodiesterases creates nanometer-size domains of low cAMP concentrations [21]. This mechanism underlines the relevance of maintaining second messengers concentration under a precise spatiotemporal control. This concept goes hand in hand with the need to have nanodomains of receptors juxtaposed with the signaling transducer (i.e., adenylyl cyclase or phospholipase C) to form patches of signalosomes on the plasma membrane. GPCR oligomerization can accomplish this role by creating receptor clusters, as it has been shown for several GPCRs [74,75,76,77]. Receptor clustering is strengthened by scaffolding proteins, and among them, caveolins. Caveolins are a family of integral membrane proteins that are the principal components of caveolae, a subset of sphingolipid/cholesterol-rich plasma membrane rafts that compartmentalize cell signaling [78]. There are three caveolin proteins, with caveolin-1 and -2 having overlapping patterns of expression throughout numerous tissues, and caveolin-3 mostly expressed in muscle and the nervous system [79]. Unlike most integral membrane proteins, caveolins adopt an unusual topography on the membrane with both the N- and C-termini lying on the cytoplasm and the hydrophobic domain forming an intramembrane loop [80]. Caveolins are particularly suited for the spatial organization of multimolecular signaling complexes, as they may act as anchors within caveolar membranes concentrating signaling molecules [81]. Two lines of evidence support the role of caveolins in signal compartmentalization. First, caveolins self-assemble to give rise to high molecular weight oligomers in the intracellular coat of caveolae, and imaging of fluorescently labeled caveolin at the plasma membrane with total internal reflection fluorescence (TIRF) reveals that they are spatially distributed in aggregates of various sizes [82]. Second, caveolins bind and regulate the activity of several signaling proteins, including components of the GPCR signaling modules, such as α-subunits of G proteins and protein kinase A [83,84]. Thus, caveolins may very well serve as organizing elements of GPCR complexes within the caveolae domains of the plasma membrane [85,86,87]. This view is supported by many studies; for instance, adrenergic β1 and β2 receptors are enriched with Gαs protein and adenylyl cyclase VI in caveolae of cardiac myocytes [88,89,90]. Another good example is how caveolin-1 acts as an organizing template for the µ-opioid receptor, Gαs protein, and adenylyl cyclase, delimiting the membrane compartment where signaling occurs in the spinal cord [91]. This role of caveolins as organizing elements that regulates cell signaling is also underpinned by the alteration of signaling when they are up or down regulated [92]. Taken together, these data suggest that oligomers of GPCRs could form the core around which the signal transduction machinery is built, and scaffolding proteins like caveolins could stabilize these signalosomes in membrane nanodomains, which have specific signaling activities. 

## 10. Novel Approaches to Study Integration of Signaling by GPCR Homo- and Heterodimers

The early works on GPCR heterodimers did not discern whether the integration of the incoming signals occurred at the receptor level, with the heterodimer enhancing the coupling efficiency to G proteins or β-arrestin, or downstream at an intracellular signaling level. These difficulties were bypassed by designing optical biosensors based on fluorescent resonance energy transfer (FRET) or bioluminescence resonance energy transfer (BRET) principles that could directly measure changes in intra-molecular receptor conformation upon ligand binding [93]. These types of Intra-FRET or Intra-BRET tools are generated by inserting two florescent proteins into different parts of the same receptor protomer, such as in the third intracellular loop and in the carboxy terminus. This allows the detection of conformational changes within the GPCR structure that elucidate kinetic and magnitude properties of GPCR activation with high temporal resolution in living cells. Importantly, the RET changes upon stimulation of both protomers of the heterodimer could be crucial for understanding the observed biochemical data that could now be interpreted as either dependent on the modifications of the coupling efficacy at the level of the GPCR heterodimer or attributed to downstream effects. The first of such reports was published by Vilardaga in 2008 [52], who demonstrated that morphine binds to the μ-opioid receptor triggering also a conformational change into the noradrenaline-bound α2A-adrenergic receptor that reduces the efficacy of noradrenaline to activate the receptor. A subsequent work by Hlavackova et al. (2012) [94] expanded this approach by examining the activation-dependent inter- and intraprotomer conformational changes in the metabotropic glutamate receptor 1 (GluR1). In a GluR1 dimer upon ligand activation, only one of the GluR1 protomer assumed an active conformation that stimulated the G-protein. In another example, Sleno et al. (2017) [95] showed that the conformational change between the two protomers of the heterodimeric AT1 angiotensin and prostaglandin F2α receptors, detected by BRET assays, was responsible for intracellular responses mediated manly via Gαq protein activation and by proximal phospholipase C signaling partners, making these proteins all together as part of a single signalosome. However, these FRET and BRET approaches have intrinsic limitations such as the low sensitivity and problems correlated to the size of the fluorescent probe attached to the receptor that can alter its activity [96]. Enhancement in fluorescent sensitivity was achieved with SNAP, CLIP, and Halo tags, which are self labelling proteins and react covalently with substrates like benzylguanine, benzylcytosine, and chloralkane, respectively, which are tagged with organic fluorophores that emit much more photons than the common fluorescent proteins [97]. Moreover, the long-lived fluorescence emission of these tags could be measured with time-resolved (TR)-FRET after the short-lived background fluorescence is abated, thus offering a powerful tool to study changes in receptor intra and intermolecular conformation [96,98]. Another huge improvement was obtained by reducing the size of the fluorescent biosensor as in the case of the fluorescein arsenical hairpin binder (FlAsH) and its red analogue ReAsH, which bind to a specific six amino acid sequence motif CCPGCC (one letter amino acid code) that could be inserted in the amino acid sequence of the targeted receptor. This sequence motif minimally modifies the structure of the receptor, and these fluorescent biosensors have a molecular weight that is below 1000 Dalton in size [99]. The sequential labeling of two different protein motifs in the receptor, CCPGCC and FLNCCPGCCMEP with FlAsH and ReAsH, respectively, allows the analysis of their interaction by colocalization and FRET with minimal disturbance to the function of the receptor at which they are attached [100].

Another interesting approach for studying GPCR dimerization processes comes from computational biology. In particular, the last decade has witnessed an exponential increase in GPCR crystal structures in which the receptor was framed in its active or inactive states and the conformational changes needed for GPCR signal transduction highlighted [101,102]. These structural data have then been used by the private and public sectors for drug discovery and more recently to identify the GPCR interfaces important to generate oligomer complexes. In fact, using algorithms and computer analysis based on these structural data it is possible to run simulations and thus predictions of compounds able to modulate the biological functions of the GPCR-target [103], but also of those GPCR structure interfaces crucial for protein–protein interactions [104,105,106,107]. In fact, although crystalized oligomers and dimers have been produced, such data do not consider membrane environments and are harder to obtained; therefore, the development of computational biology approaches able to run structure-based predictions of GPCR dimerization might be proven to be an invaluable asset to study these complex protein–protein interactions. However, this approach has its limitations and predictions of GPCR oligomer interfaces needs to be challenged by experimental testing [108]. It is worth noticing that developing more and more tools to design drugs to interact solely or preferentially with GPCR higher complex structures would be an important step forward for the development of new therapeutics that have the potential to revolutionize GPCR pharmacology.

Likely, these new methods of analysis will soon allow not only to dig deeper into the mechanisms of GPCR dimerization but also to improve our understanding on how GPCR heterodimerization modifies the coupling properties of the receptor protomers for G-proteins or other interacting partners and to pharmacologically target these complexes and develop better drugs.

## 11. Concluding Remarks

As we have shown in this review, interaction of receptors at the plasma–membrane plays an important role in the integration of cell signaling. In order to communicate, cells need to distinguish relevant signals from the background, and to filter this information at different levels. At the plasma membrane GPCRs interact canonically with numerous proteins such as G-proteins and β-arrestins, but they can also interact with other receptors by forming homo- and heterodimers. Furthermore, they can also form complexes on the plasma membrane with many receptors and proteins to generate higher-order oligomers. Importantly, these protein interactions at the membrane levels might result in an attenuation, or augmentation, or even a change in the type of intracellular signaling triggered by specific stimuli or a combination of them. Moreover, besides these information filtering mechanisms, another important modulation of extracellular signaling occurs intracellularly where second messengers are finally localized into specific areas within the cell by proteins either able to bind or degrade them. Evolution has shaped membrane receptors in a way to interact with each other hundreds of million years ago as showed by the functional dimerization of the yeast α-factor receptors [59]. The need of such an abundance of interactions between GPCRs themselves and with receptors belonging to other families such as ligand-gated ion channels and tyrosine kinase receptors is exemplified by what occurs in neurons. Neurons form complex signaling networks and each neuron must arrange hundreds of incessantly external incoming messages. In such a scenario, integration of signaling downstream to the plasma membrane will increase the chance to blurry the relevant information, while integration of the signaling at the plasma membrane will consent to retain most of the information. From a pharmacological point of view, receptor heterodimerization constitutes an un-precedented opportunity for drug discovery, for example, with bivalent drugs [109] or allosteric compounds [110], which might be able to specifically target tissues, limiting side effects and promoting better therapeutic effects.

## Data Availability

Not applicable.
